# Antioxidant responses of *Triticum aestivum* plants to petroleum-derived substances

**DOI:** 10.1007/s10646-018-1988-3

**Published:** 2018-10-11

**Authors:** Milena Rusin, Janina Gospodarek, Gabriela Barczyk, Aleksandra Nadgórska-Socha

**Affiliations:** 1 Department of Agricultural Environment Protection, University of Agriculture, al. A. Mickiewicza 21, 31-120 Krakow, Poland; 2 Department of Ecology, University of Silesia in Katowice, Bankowa 9, 40-007 Katowice, Poland

**Keywords:** Soil contamination, Antioxidants, Antioxidant, Enzymes, *Triticum aestivum*

## Abstract

Winter common wheat (*Triticum aestivum* L.) plants were cultivated on petroleum products contaminated soils with and without using biopreparation ZB-01. We determined the impact of soil contamination with petrol, diesel fuel and engine oil on selected antioxidant enzymes and the levels of antioxidants in the leaves of winter wheat. The impact of petroleum products on selected morphological characteristics of the plants, levels of nutrients and heavy metals was also assessed. Winter wheat was relatively resistant to soil contamination with petroleum products, and did not show a significant impact on the morphological characteristics of the plants. The levels of nutrients and heavy metals in the plants depended on the type of pollutant and the analyzed component.‬ Biopreparation ZB-01 generally resulted in an increase in calcium levels in the plants.‬ The winter wheat plants growing in soil contaminated with engine oil were characterized by higher levels of zinc, lead, manganese and cadmium than the control plants.‬ Biopreparation applied to the soil contaminated with petrol resulted in a slight increase in the levels of lead and zinc in the plants.‬ The petroleum products affected the activity of antioxidant enzymes and the levels of antioxidants in the plants.‬ The general markers of soil contaminated with diesel fuel and petrol were POD activity and proline levels. Use of the ZB-01 biopreparation caused an increase in the levels of proline and -SH groups and an increase in the levels of carbon and calcium in the plants and had no effect on the morphological characteristics of plants.‬

## Introduction

One of the main problems of the natural environment is pollution with petroleum products, associated mainly with the operations of the petrochemical industry (Jørgensen et al. 2000; Das and Chandram 2011). Accidental release of these products into the environment is dangerous because of the mutagenic and carcinogenic properties of their components (Krahl et al. 2002; Jain et al. 2011). After reaching the soil, petroleum products destroy its structure, disrupt the air-water balance (Iturbe et al. 2007), alter its physicochemical properties (Caravaca and Rodán 2003), affect the biological balance (Baran et al. 2004; Labud et al. 2007), disrupt soil enzymatic activity (Wyszkowska et al. 2002) and have a negative impact on terrestrial and soil mesofauna (Gospodarek 2008; Rusin and Gospodarek 2016; Gospodarek et al. 2016). Petroleum products also have a negative impact on the growth and development of plants (Ogboghodo et al. 2004b; Shirdam et al. 2008; Gbadebo and Adenuga 2012), change the levels of macro and microelements in plant organs (Wyszkowski and Ziółkowska 2009a, Wyszkowski and Ziółkowska 2009b; Shukry et al. 2013), and modify the levels of heavy metals in them (Ujowundu et al. 2011; Rusin et al.‬ 2015).‬

Petroleum products, and above all the heavy metals they contain, can also cause oxidative stress in plants resulting from an over production of reactive oxygen species (ROS) and a lack of respective neutralization (Pawlak-Sprada et al. 2011). Through reactions with many cellular structures (proteins, membranes, nucleic acids), ROS cause undesirable changes in cells which may contribute to numerous metabolic disorders. An important role in the fight against ROS is played by antioxidative mechanisms in plants. Information on changes in the activity of antioxidative enzymes such as superoxide dismutase (SOD), catalase (CAT), guaiacol peroxidase (POD), and antioxidant levels (proline, non-protein -SH groups), may indicate their usefulness as biomarkers and also allow a better understanding of the defense mechanisms of plants exposed to oxidative stress (Nadgórska-Socha et al. 2013b).‬

Physical and chemical methods used to remove petroleum products from the soil are generally expensive and have limited effectiveness, yet may be replaced by biological methods that rely on the use of living organisms to remove harmful xenobiotics from the soil (Meintanis et al. 2006; Haritash and Kaushik 2009; Milić et al. 2009). Microorganisms use petroleum products as a source of energy, and the final effect of bioremediation is the transformation of these, their stabilization and transformation into non-toxic compounds such as carbon dioxide and water (Milić et al. 2009; Millioli et al.‬ 2009). Bioremediation allows a significant degradation of petroleum hydrocarbons in a short period of time, with a reduction in their levels in the soil (Dindar et al. 2013).‬

The aim of the conducted research was to determine the impact of soil contamination with selected petroleum products, such as petrol, diesel fuel and used engine oil, on selected antioxidant enzymes and the levels of antioxidants in the leaves of winter common wheat (*Triticum aestivum* L.). In addition, we investigated the impact of these petroleum products on selected morphological characteristics of the plants, levels of nutrients and heavy metals, as well as selected physical and chemical properties of the soil. We also evaluated the influence of bioremediation by using biopreparation ZB-01 on all the aforementioned parameters.‬

## Material and methods

### Experimental setup

The field study area was in Experimental Station of the University of Agriculture in Krakow, located in Mydlniki near Krakow (Poland; 50.0815°N, 19.84730°E). In November 2009, indigenous soil (loamy-sand) (detailed characteristics of the soil was given in a previous paper (Gospodarek et al. 2016), other parameters in Table 1) was placed in special containers of 1 m^3^ volume, retaining the natural arrangement of layers. The containers were sunk in the ground so that their upper edge was at the same level as the surface of the soil. The description of the containers and its arrangement in the experimental field were presented in our previous paper (Gospodarek et al. 2016). The soil in the containers was left for 8 months without any intervention in order to regain its natural biological functions. In June 2010, the soil surface was artificially contaminated with petrol (P), used engine oil (EO), and diesel oil (DF) in a quantity of 6000 mg of petroleum product per 1 kg of soil dry mass in the container (i.e. typical for medium-contaminated soils), by pouring it on the soil. The non-contaminated soil in identical containers served as a control. Petrol (BP Unleaded 95) and diesel fuel (BP Diesel Fuel) came from BP petrol station whereas engine oil (PLATINUM Classic Semisynthetic 10W-40) from Orlen petrol station. Engine oil was used for one year (in a petrol engine) prior to applying in this experiment. It was homogeneous quality. It came from one tank in which was collected in the amount needed to use in the experiment. Before use, it was mixed thoroughly. Used petroleum products were the same as described in our previous paper (Rusin et al. 2017). After 1 week, soil in half of the number of containers was subjected to the bioremediation by adding biopreparation ZB-01. The biopreparation contained selected prokaryotic organisms, which were isolated over years from sites heavily polluted with organic compounds. It consisted mainly bacteria: *Stenotrophomonas*, *Pseudomonas*, *Moraxella*, *Acinetobacter*, *Alcaligenes*, *Ochrobactrum, Comamonas, Burkholderia, Corynebacterium*, *Oligella* and was specially prepared for this experiment in Biochemistry Department of University of Agriculture in Kraków. The ZB-01 treatment was proceeded by fertilization with the use of “Azofoska” compound fertilizer. The way of ZB-01 and “Azofoska” application were described in our previous paper (Gospodarek et al. 2016). The usefulness of the proposed ZB-01 microbial biopreparation in a remediation of soils contaminated with petroleum products was confirmed earlier (Petryszak et al. 2008; Kaszycki et al. 2014; Gospodarek et al. 2016). After 1 year, the bioremediation treatment was repeated. In 3 subsequent years, the soil in the containers was left undisturbed. The experiment was established in four replications in line with the randomised blocks method.

**Table 1:** The effect of petroleum products on selected physico-chemical soil properties‬

Treatment	Bulk density (g cm^−3^)	Soil moisture (%)	Dry matter (%)	pH in H_2_O	pH in KCl	CaCO_3_ (%)	K_2_O (mg 100 g^−1^)	P_2_O_5_ (mg 100 g^−1^)	C_total_ (%)	N (%)
Initial soil	–	–	–	7.12	6.45	0.17	17.17	16.37	1.04	0.09
EO 0 R	1.172	13.48	86.52	6.01	4.95	0.08	19.13	9.19	1.70	0.11
EO R	1.231	13.48	86.52	6.24	5.45	0.08	14.66	7.76	1.44	0.09
DF 0 R	1.253	13.98	86.02	6.16	4.93	0.17	18.41	15.89	1.17	0.10
DF R	1.257	13.22	86.78	6.23	5.26	0.17	15.89	12.02	1.14	0.09
P 0 R	1.273	12.37	87.63	6.65	5.85	0.34	12.77	14.36	0.93	0.08
P R	1.329	13.45	86.55	6.85	5.78	0.25	17.86	13.44	1.00	0.09
C 0 R	1.217	12.38	87.62	6.98	6.12	0.34	13.00	11.85	0.97	0.09
C R	1.250	13.14	86.86	6.33	5.10	0.34	13.67	11.52	0.97	0.09

*EO* soil contaminated with engine oil, *DF* soil contaminated with diesel fuel, *P* soil contaminated with petrol, *C* control soil, *0* *R* without bioremediation, *R* with bioremediation

### Soil

Physicochemical analyses of the initial soil and 4 years from the moment of contamination were carried out at the Soil Laboratory of the Department of the Forest Management Office in Kraków.‬ Granulometric composition was determined using sieves and the aerometric method with dry fractionation according to PN_R_04032: 1998, bulk density – by weight method according to PB-06, ed.4, dry matter levels and soil moisture – according to PN-EN 13040:2009, pH in H_2_O and KCl – by potentiometric method, carbonate levels – by Scheibler method according to PB-05, ed.3, bioavailable potassium – FAAS method according to PN-R-04022: 1996 + Azl: 2002, available phosphorus – by spectrophotometric method according to PN-R-04023:1996, total carbon – by high-temperature combustion method with IR detection according to PN-ISO 10694:2002 (for initial soil according to PB-01, ed.4), nitrogen – by titration method according to PN-ISO 11261:2002 (for initial soil by Kjeldhal’s method according to PB-02, ed. 5).‬ After 1 month from soil contamination, analysis of the total petroleum hydrocarbons (TPH) in the soil was performed. The content of TPH in the soil contaminated with EO was over 36000 mg kg^−1^ dry matter, DF – over 13000 mg kg^−1^ dry matter, P – about 2700 mg kg^−1^ dry matter, in control soil – about 3500 mg kg^−1^ dry matter (Gospodarek et al. 2016). In order to monitor changes in the levels of petroleum products in the experimental soils four years after soil contamination, analyses concerned petroleum hydrocarbons, divided into gasoline range hydrocarbons (C_6_–C_12_ hydrocarbons according to ISO 22155:2013) and mineral oil hydrocarbons (C_12_–C_36_ in accordance with the PN-EN ISO 16703:2011 standard) were conducted. These analyses were performed at the Laboratory of Physical-Chemical Analyses, WESSLING Polska sp. z o.o. in Kraków, Poland.

### Plants

In the middle of October 2013, winter common wheat seeds of the Batuta variety were sown on prepared and fertilised soil in containers, at 400 seeds per container (according to the seed sowing standard). Pre-sowing fertilization with Azofoska was applied, introducing 5.44 g N, 2.56 g P_2_O_5_ and 7.64 g K_2_O per container (the dose was determined on the basis of fertilizer manufacturer’s recommendations). In all the containers, around 90% of wheat plants survived winter.

### Plant growth

Evaluation of plant morphology and crop structure elements was performed on fully matured wheat plants.‬ We randomly selected 15 plants from each container to determine plant height, weight of the ear, weight of straw and weight of grains from the ear.‬

### Analysis of the biochemical parameters of the plants

In order to determine antioxidant enzyme the plant samples (20 randomly selected plants from each container) were taken during the flowering stage, the highest metabolic point during the plant life cycle (Dazy et al. 2008). The analyses of enzymes activity as well as proline, non-protein thiols and protein content were previously described in details (Nadgórska-Socha et al. 2013a, 2013b). Crushed plant parts (leaves) were homogenized in a 100 mM phosphate buffer (pH 6.8) for POD and 50 mM (K/Na) phosphate buffer for CAT at 4 °C and centrifuged at 12,000 g for 20 min. The supernatant was used to determine the enzyme activity levels. The catalase activity was determined according to Aebi (1984). The catalase activity was expressed in μmol consumed H_2_O_2_ min^−1^mg protein^−1^. The analysis of superoxide dismutase (SOD) was performed according to Beauchamp and Fridovich method Beauchamp and Fridovich (1971) and activity was expressed in U. The total protein was estimated using the Bradford (1976) method. To measure the contents of non-protein thiols it was used method of Mass et al. (1987). The acid–ninhydrin method was used to determine the proline content. The proline content was calculated as described by Bates et al. (1973), expressed in micromoles proline per gram fresh weight.

### Analysis of element concentration in plants samples

In order to determine the macroelement (Ca, Mg, K, N, S, C) and trace element (Fe, Zn, Pb, Mn, Cd, Cu, Ni) concentrations in plants’ samples (20 randomly selected plants from each container), plant material (aboveground parts) was washed in tap, next in distilled water and dried at 105 °C [previously described in detail (Nadgórska-Socha et al. 2013a)]. Dry weight subsamples 0.25 g were wet digested in concentrated HNO_3_ at the maximum of 120 °C and then diluted to 25 mL with deionized water (Lin et al. 2008). Trace elements and macroelement (Ca, Mg, K) contents were measured using flame absorption spectrometry (Thermo Scientific iCE 3500) (Nadgórska-Socha et al. 2013a). Carbon, nitrogen and sulphur contents were determined in a Variomax CNS analyzer. The accumulation of nutrient elements and trace elements (ANE and ATE) in the studied plants was analyzed according to Ostrowska and Porębska method Ostrowska and Porębska 2002 and described in details in Nadgórska-Socha et al. (2015). In order to calculate the sum of the elements, the values describing the amount of each element were converted into equivalents. The sum of elements was calculated according to the following formula:

$$Y = \mathop {\sum}\nolimits_{i = 1}^i {\frac{Z}{z}}$$

Z = element content in mg kg^−1^, and z is the atomic mass/valence of ion

After the estimation of the sum of accumulation, a percentage of each element (X) should be calculated:

$$X = \frac{{\frac{Z}{z} \times 100}}{Y}$$

### Statistical analysis

The obtained results were analyzed, checked for normality (Shapiro–Wilk test with Lilliefors correction) and equality of variance (Levene’s test). The significance of differences between the means were tested by two-factor variance analysis (STATISTICA 10.0 software), and the means were differentiated by Fisher’s LSD test at *p* < 0.05. CANOCO 4.5 was used to carry out Principal Component Analysis (PCA) (Ter Braak and Šmilauer 2002). Principal Component Analysis assessed the similarities and relations between biochemical parameters and elemental content in the plants. The data were log transformed Y = log(Y + 1).

## Results

### Soil

Compared to the initial soil, the control soil (after 4 years) was characterized by a slight decrease in pH and lower levels of available potassium and phosphorus (by more than 4 mg 100 g^−1^ of soil), while the levels of carbonates increased (Table 1). The levels of total carbon and nitrogen in both cases were similar. After 4 years from the time of contamination of the experimental soil, the petroleum products used did not significantly affect its bulk density, moisture, dry matter and total nitrogen levels.‬ However, they caused an increase in soil acidification, which was particularly evident in the case of the soil contaminated with engine oil, in which the pH value in H_2_O was nearly 1 scale lower than in the control. Both engine oil and diesel fuel led to a decrease in the levels of available phosphorus in the soil, and also to an increase in the levels of available potassium and total carbon compared to the initial soil.‬ Petrol contributed to a decrease in the levels of available potassium and phosphorus, and also caused a two-fold increase in the levels of carbonates in the soil.‬

The use of ZB-01 resulted in a slight increase in bulk soil density in all analyzed objects, as well as usually contributed to an increase in pH (except for the control in which the pH value in H_2_O decreased by 0.65 after biopreparation). In addition, the soils contaminated with engine oil and diesel fuel and subjected to bioremediation were characterized by a lower level of available potassium and phosphorus and total carbon than the soils for which the biopreparation was not used. In the case of soil contaminated with petrol, a decrease in the levels of carbonates and available phosphorus was noted, while the levels of total carbon and available potassium increased following the use of ZB-01. In the control treatment, the biopreparation did not visibly affect most of the physico-chemical properties of the soil.‬

The levels of C_6_–C_12_ hydrocarbons after 4 years were less than 0.8 mg kg^−1^ in all the analyzed soils (Table 2). The levels of C_12_–C_36_ hydrocarbons in the control soil both with the use of ZB-01 biopreparation as well as without its use were lower than 6 mg kg^−1^. The largest amount of C_12_–C_36_ hydrocarbons was recorded in the soil polluted with engine oil, almost twice less in the diesel-contaminated soil and more than three times less in the petrol-contaminated soil. The used biopreparation caused a significant decrease in C_12_–C_36_ hydrocarbons in all contaminated soils (about 2 times in the case of EO and P and over 3 times in the case of DF).‬

**Table 2:** The levels of petroleum products in the soil after four years from contamination (mg kg^−1^)

Treatment	Gasoline range hydrocarbons (C_6_–C_12_)	Mineral oil hydrocarbons C_12_-C_36_)
EO 0 R	<0.8	1000
EO R	<0.8	530
DF 0 R	<0.8	750
DF R	<0.8	210
P 0 R	<0.8	12
P R	<0.8	7
C 0 R	<0.8	<6
C R	<0.8	<6

Symbols as in Table 1‬

### Plant growth

Plants growing in the soils that had been contaminated with engine oil and diesel fuel four years earlier, were significantly lower than the control plants and those growing in soil contaminated with petrol (Table 3).‬ The use of the ZB-01 biopreparation in soil contaminated with petrol caused an increase in plant height by more than 5 cm compared to the soil in which the biopreparation was not used. Increase in height to the level of the control plants was also obtained in the case of the soil contaminated with DF. However, there was no significant influence by the petroleum products and biopreparation on other morphological features of winter common wheat.‬

**Table 3:** The effect of petroleum products on the growth of *Triticum aestivum* L

Treatment	Height of plant (cm)	Weight of ear (g)	Weight of straw (g)	Weight of grains from the ear (g)
EO 0 R	60.30 (±5.9) ^a,*^	2.50 (±0.2) ^a^	1.21 (±0.1) ^a^	1.96 (±0.1) ^a^
EO R	60.80 (±5.3) ^a^	2.44 (±0.2) ^a^	1.23 (±0.1) ^ab^	1.89 (±0.2) ^a^
DF 0 R	61.03 (±8.0) ^ab^	2.58 (±0.5) ^a^	1.25 (±0.1) ^ab^	1.99 (±0.4) ^a^
DF R	63.68 (±6.3) ^bc^	2.56 (±0.4) ^a^	1.27 (±0.1) ^ab^	2.00 (±0.3) ^a^
P 0 R	64.05 (±7.4) ^c^	2.75 (±0.2) ^a^	1.32 (±0.1) ^ab^	2.20 (±0.2) ^a^
P R	69.55 (±6.4) ^d^	2.69 (±0.1) ^a^	1.42 (±0.2) ^b^	2.18 (±0.1) ^a^
C 0 R	63.70 (±7.5) ^c^	2.68 (±0.4) ^a^	1.33 (±0.1) ^ab^	2.11 (±0.3) ^a^
C R	64.73 (±3.8) ^c^	2.65 (±0.6) ^a^	1.35 (±0.2) ^ab^	2.10 (±0.5) ^a^

^*^ Means (±SE) in columns marked with the same letters do not differ according to the LSD test at *p* < 0.05.‬ Symbols as in Table 1

### Analysis of the biochemical parameters of the plants

**Fig. 1:**
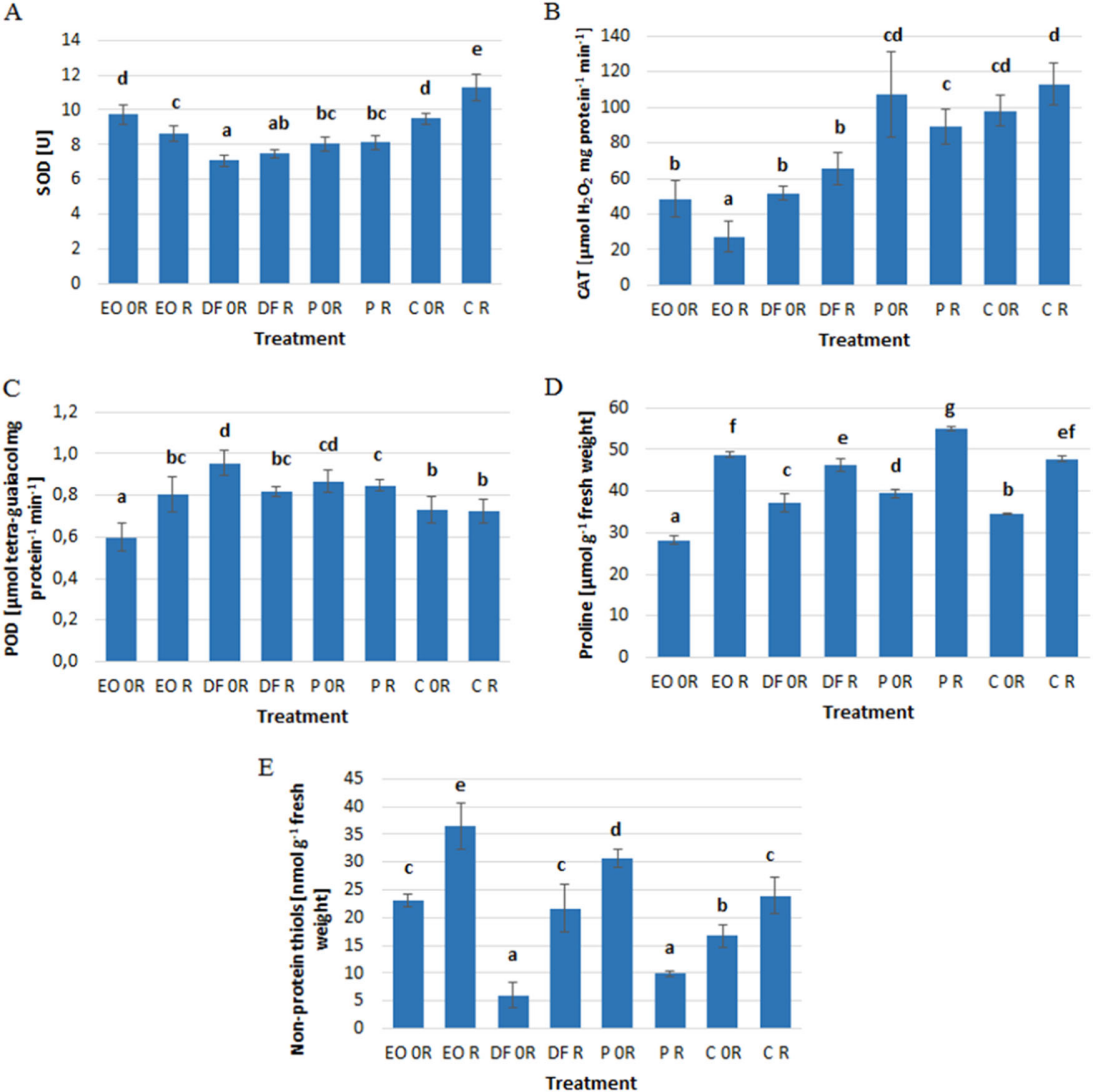
The effect of petroleum products on the activity of antioxidant enzymes. **a** superoxide dismutase. **b** catalase. **c** guaiacol peroxidase, and antioxidants. **d** proline. **e** non-protein thiols in winter wheat leaves. Values marked with the same letters do not differ significantly according to LSD test at *p* < 0.05. Vertical bars mean SE. Symbols as in Table 1

Engine oil caused a significant decrease in the activity of catalase and peroxidase and a lowering of proline levels in winter wheat plants, but on the other hand also contributed to an increase in non-protein thiol groups (Fig. 1b–e). Plants growing on soil contaminated with diesel fuel were characterized by a lower activity of superoxide dismutase, catalase and lower levels of -SH groups, but also increased peroxidase activity and proline levels (Fig. 1a–e). Petrol contamination resulted in an increase in peroxidase activity, proline levels and -SH groups, while it caused a significant reduction in superoxide dismutase activity (Fig. 1a, c–e).

Supporting the bioremediation process with the use of ZB-01 in all analyzed soils resulted in a significant increase in proline levels and non-protein thiol groups (except for the soil contaminated with petrol) (Fig. 1d, e).‬ In the soil contaminated with engine oil, ZB-01 biopreparation contributed to a decrease in the activity of superoxide dismutase and catalase (Fig. 1a, b). In the case of peroxidase, however, the use of ZB-01 resulted in reducing the differences in the contaminated soil with engine oil and diesel fuel to a level similar to that found in uncontaminated soil conditions (Fig. 1c). In other objects (i.e.‬ P and C) the biopreparation did not generally affect the activity of the tested antioxidative enzymes in the winter wheat plants.‬

### Analysis of element concentration in plants samples

**Fig. 2:**
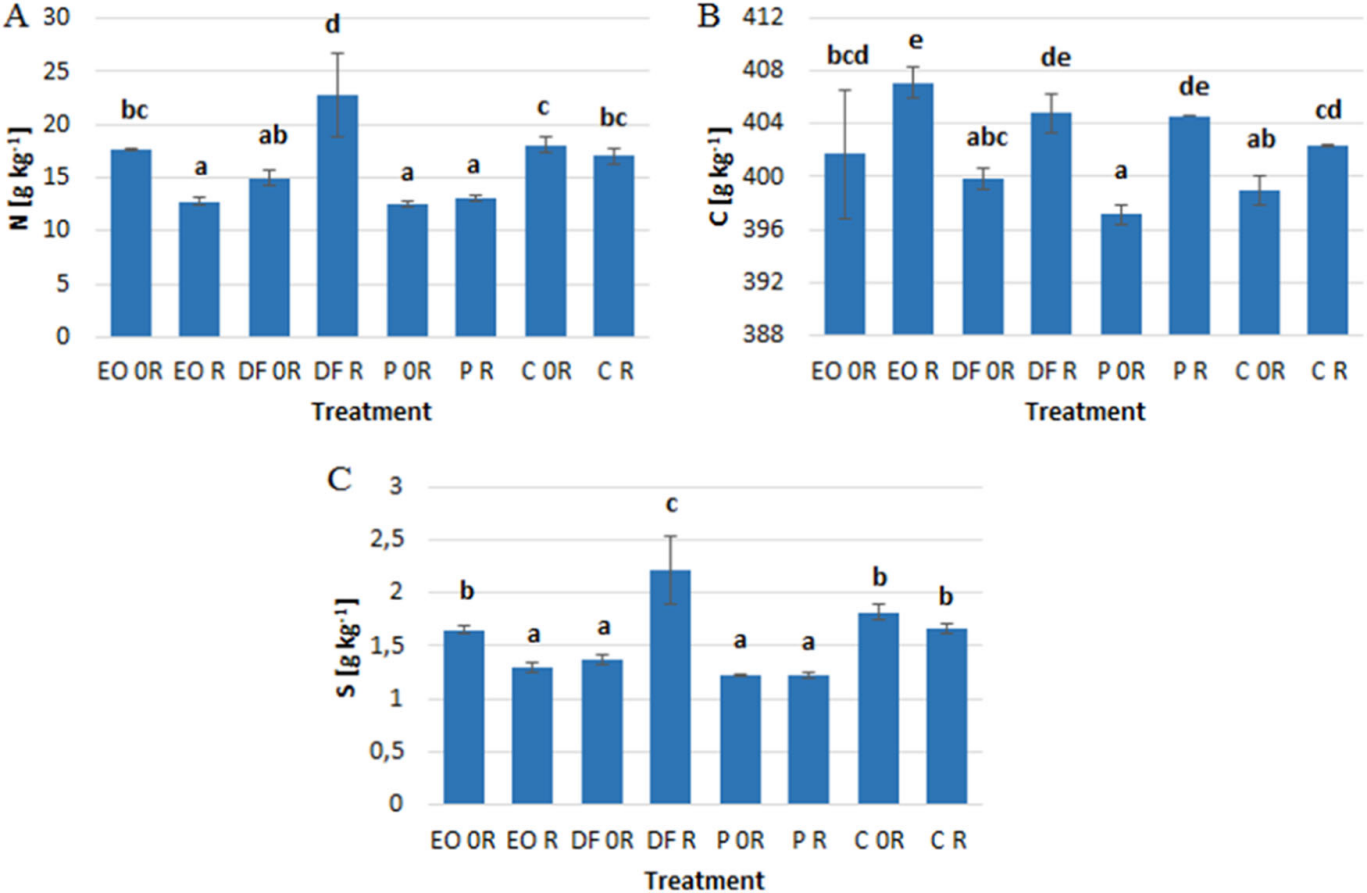
The effect of petroleum products on the levels of **a** nitrogen, **b** carbon and, **c** sulfur in the aboveground parts of *Triticum aestivum* L. (g kg^−1^)‬. Values marked with the same letters do not differ significantly according to LSD test at *p* < 0.05. Vertical bars mean SE. Symbols as in Table 1

Both diesel fuel and petrol caused a significant reduction in nitrogen and sulfur levels in the aboveground parts of winter wheat, but had no significant effect on the carbon level (Fig. 2a–c). No effect of engine oil on the levels of N, C and S was observed. Biopreparation ZB-01 applied to the soils contaminated with petroleum products and control soil led to an increase in carbon levels in aboveground parts of the plants (Fig. 2b).‬ In addition, when ZB-01 was applied to the soil contaminated with diesel fuel, an increase in nitrogen and sulfur levels was noted (Fig. 2a, c). The opposite effect was found in the soil contaminated with engine oil, where the levels of N and S dropped.‬

**Fig. 3:**
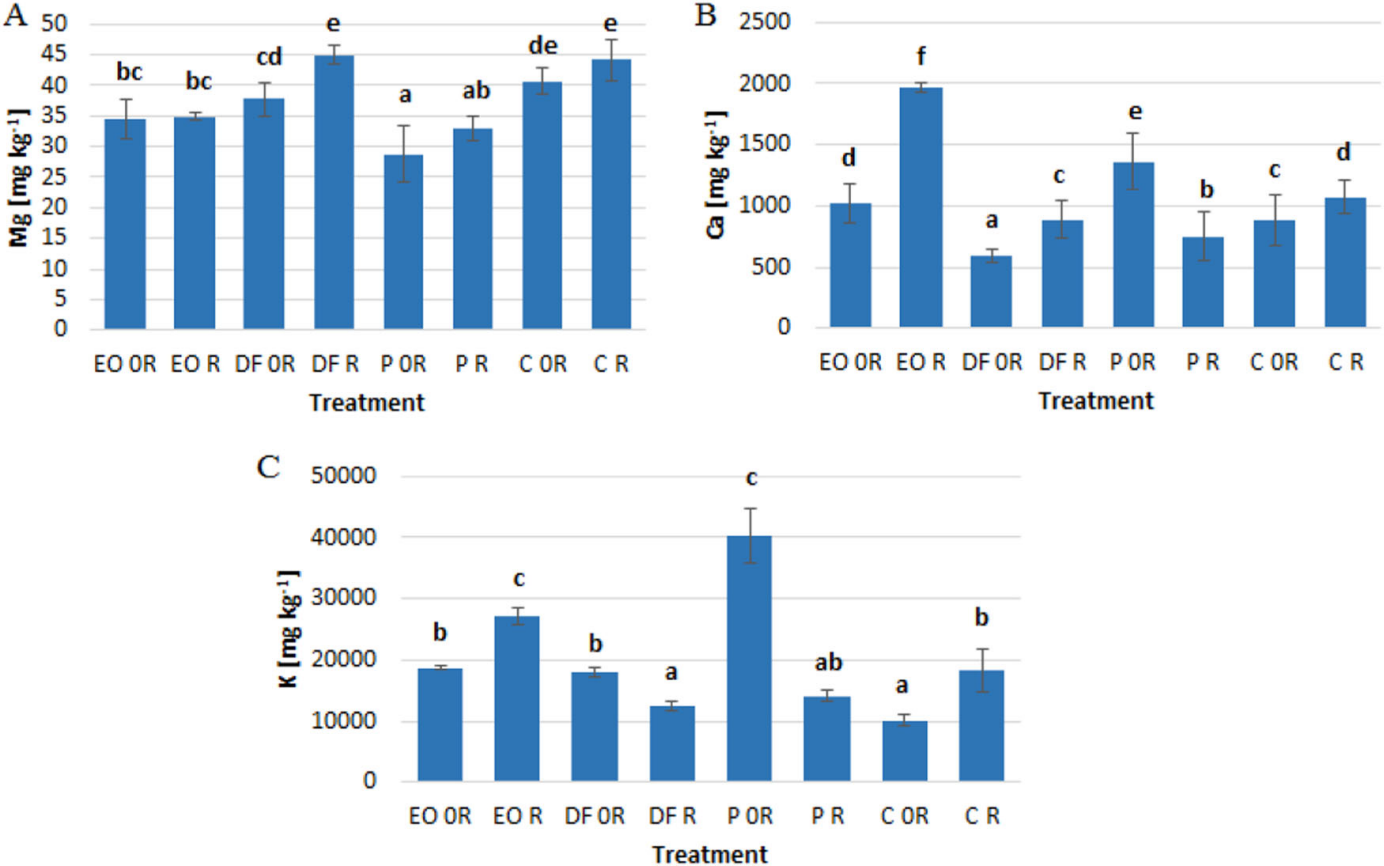
The effect of petroleum products on the levels of **a** magnesium, **b** calcium and, **c** potassium in the aboveground parts of *Triticum aestivum* L. (mg kg^−1^)‬. Values marked with the same letters do not differ significantly according to LSD test at *p* < 0.05. Vertical bars mean SE. Symbols as in Table 1

All used petroleum products increased the potassium levels in aboveground parts of winter wheat (Fig. 3c).‬ Contamination of the soil with engine oil led to an increase in calcium levels and a reduction in magnesium levels compared to the control (Fig. 3a, b). Plants growing on soil contaminated with diesel fuel were characterized by lower levels of calcium than control plants (Fig. 3b).‬ Petrol fuel in the soil caused a decrease in magnesium levels, and an increase in calcium levels in aboveground parts of winter wheat (Fig. 3a, b). Biopreparation ZB-01 generally resulted in an increase in calcium levels in the plants apart from the soil contaminated with petrol where it led to a decrease in Ca level. (Fig. 3b). When applied to the soil contaminated with engine oil and to the control soil, ZB-01 caused an increase in potassium levels in the plants, but a reverse relation was noted in the plants growing in the soils contaminated with diesel fuel and petrol (Fig. 3a).

**Fig. 4:**
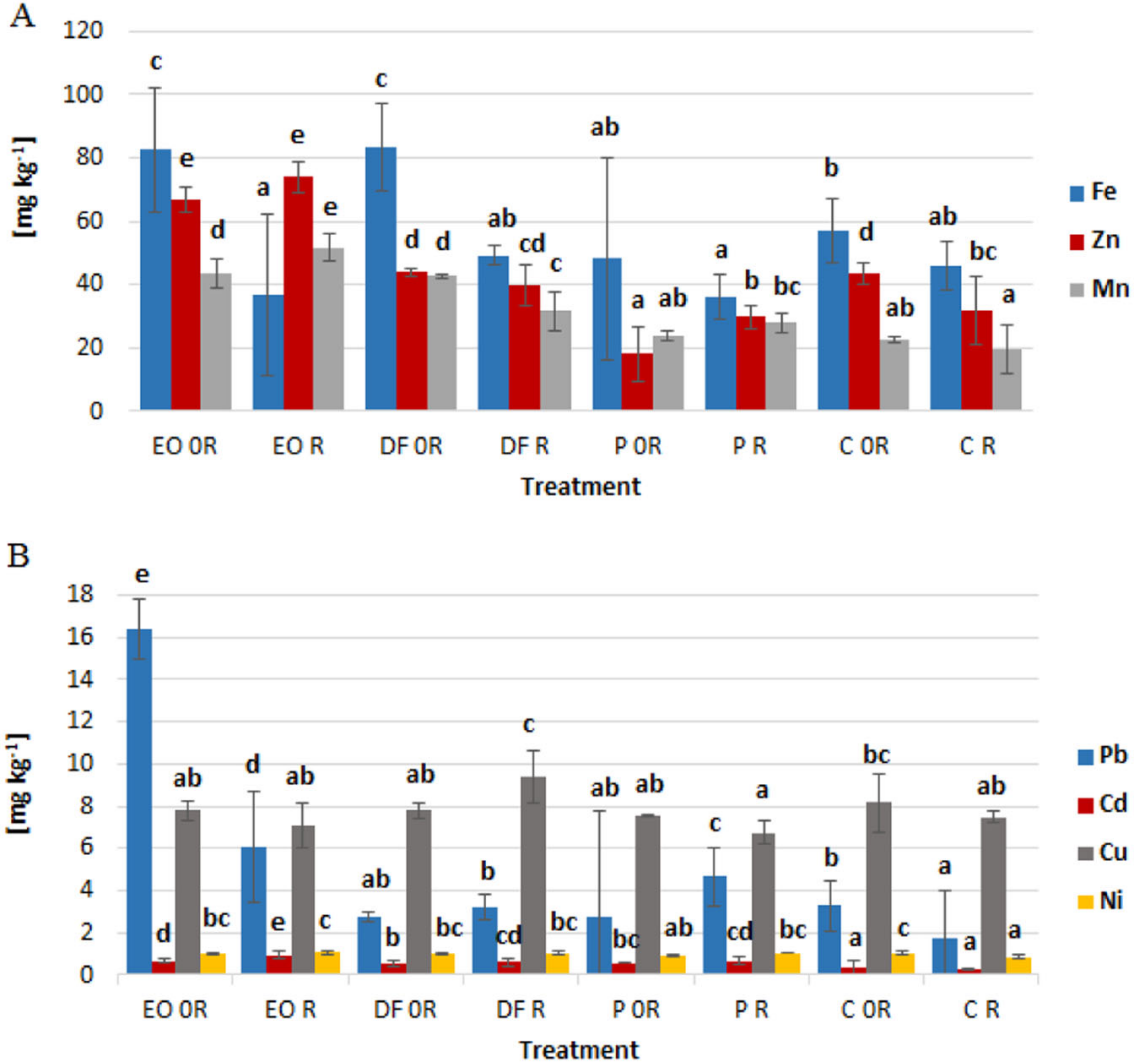
The effect of petroleum products on the levels of **a** iron, zinc, manganese, **b** lead, cadmium, cooper, nickel in aboveground parts of *Triticum aestivum* L. (mg kg ^−1^)‬. Values marked with the same letters for each element separately do not differ significantly according to LSD test at *p* < 0.05. Vertical bars mean SE. Symbols as in Table 1

Iron levels in winter wheat plants cultivated on soils contaminated with engine oil and diesel fuel were higher than in the control plants.‬ (Fig. 4a). The biopreparation in this case showed an effect offsetting this increase.‬ The winter wheat plants growing in soil contaminated with engine oil were characterized by higher levels of zinc, lead, manganese and cadmium than the control plants (Fig. 4a, b). Especially high differences were noted for lead – 5 times higher levels than in the control plants.‬ Diesel fuel, in turn, caused a nearly 2-fold increase in manganese levels and a 1.5-fold increase in the case of cadmium in the aboveground parts of the plants. Similarly, petrol led to an increase in cadmium levels; however, in this case plant tissues contained more than 2 times less zinc than in the control plants.‬ Copper levels showed the lowest variability in the wheat plants exposed to petroleum products (Fig. 4a, b)‬.

Biopreparation applied to the soil contaminated with petrol resulted in a slight increase in the levels of lead and zinc in the plants (Fig. 4a, b).‬ At the same time, these metals, as well as Ni, occurred in lower concentrations in control plant tissues after biopreparation was applied to this soil.‬ In the soils contaminated with engine oil and diesel fuel, the use of the biopreparation contributed to a slight increase in the levels of cadmium, as well as manganese (only EO) and copper (only DF), and a decrease in the levels of lead (only EO) and manganese (only DF).

Increased concentration of trace elements was confirmed by a higher accumulation trace element (ATE) index (Table 4).‬ ATE was higher in plants exposed to EO and DF (with and without remediation) in comparison to ATE calculated for the control plants. There was also a higher percentage of Pb, Cd, Zn and Mn in the plants exposed to petroleum products. The accumulation of nutritional elements (ANE) index (Table 8) was higher in plants grown in soils contaminated with petroleum products, such as as DF and EO. In the plants cultivated in soils with ZB-01 we found a reduced ANE. ‬

**Table 4:** ATE and ANE (accumulation of trace elements and nutrient) in the studied plants

Treatment	ATE trace elements [mmol_c_ kg^−1^]	ANE macroelements [mmol_c_ kg^−1^]	Zn [%]	Pb [%]	Fe [%]	Mn [%]	Cd [%]	Mg [%]	Ca [%]	Cu [%]	K [%]	Ni [%]	S [%]	N [%]
EO 0 R	7.02	3335.03	29.02	2.25	42.10	22.47	0.17	0.09	1.53	3.50	14.31	0.48	19.96	64.11
EO R	5.80	3535.53	39.04	1.02	22.75	32.45	0.29	0.08	2.77	3.83	19.67	0.62	16.66	60.81
DF 0 R	6.21	3152.97	21.67	0.43	48.19	25.08	0.15	0.10	0.94	3.94	14.60	0.54	14.81	69.55
DF R	4.51	3180.90	27.03	0.68	39.11	25.62	0.25	0.12	1.39	6.54	10.08	0.78	19.14	69.27
P 0 R	3.45	3912.76	16.06	0.76	49.97	25.10	0.29	0.06	1.73	6.90	26.38	0.92	12.75	59.08
P R	3.53	3063.82	25.79	1.28	36.70	28.87	0.33	0.09	1.22	6.02	11.83	1.01	17.08	69.79
C 0 R	4.54	3180.94	29.38	0.69	45.14	18.20	0.14	0.11	1.39	5.65	8.12	0.79	18.58	71.81
C R	3.61	3959.33	26.85	0.47	45.73	19.50	0.12	0.09	1.35	6.51	11.84	0.81	23.44	63.27

Symbols as in Table 1

**Fig. 5:**
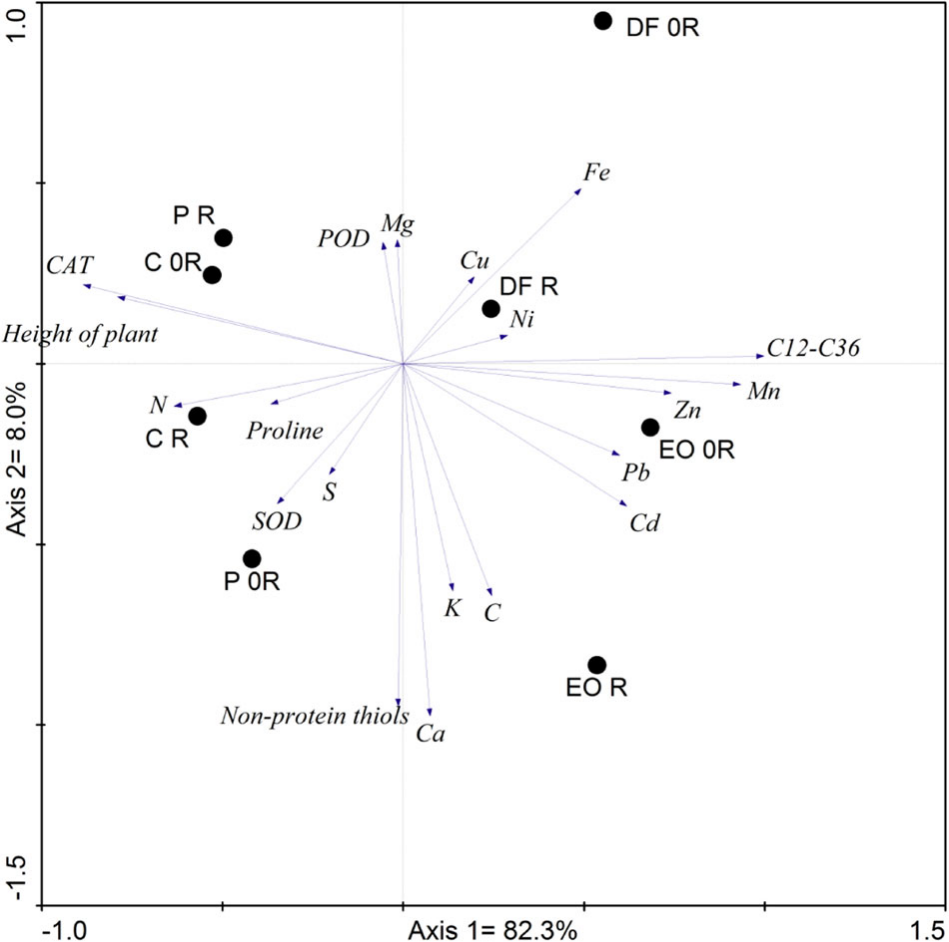
Principal component analysis performed on wheat biochemical parameters and element contents in the plants in soils treated with the investigated petroleum products

In order to show the relationship between the analyzed parameters, principal component analysis (PCA) was performed (Fig. 5).‬ Fig. 5 shows the first two principal components that together accounted for 90.3% of variance.‬ The strongest correlation with the first axis was shown by catalase and proline activity, height of plant, and N levels (positive correlation) as well as the levels of the C_12_–C_36_ hydrocarbons, Mn, Zn and Ni (negative correlation).‬ The second axis positively correlated with POD and Mg and negatively with non-protein thiols, Ca, K and C. The analysis showed large differences between the sets of samples: DF and EO (set 1) and P and C (set 2) and the impact of the bioremediation on the tested parameters in the samples contaminated with DF and EO.‬

## Discussion

Petrols and diesel oils from various manufacturers have a similar chemical composition and therefore, obtained effects can also be related to the effects of other petroleum products. Differences may occur in the case of engine oil, because we distinguish mineral, synthetic or semisynthetic oils. In our experiment we used semisynthetic oil. On the basis of the conducted tests, it was found that both engine oil and diesel fuel contributed to an increase in the total carbon levels and nitrogen levels in the soil.‬ Similar patterns have been reported by many authors (Caravaca and Rodán 2003; Riffaldi et al. 2006; Okonokhua et al. 2007). Changes in the levels of the analyzed macronutrients due to the presence of petroleum products in the soil are mainly related to their chemical structure.‬ Crude oil and petroleum products consist primarily of carbon, hydrogen, sulfur, nitrogen and oxygen (Riffaldi et al. 2006; Wyszkowski and Sivitskaya 2012), which may cause an increase in the levels of these elements in contaminated soils. However, contamination with petroleum products may also cause a decrease in the levels of nitrogen and phosphorus in the soil, which is caused by the use of these elements in the process of natural biodegradation of xenobiotics (Obire and Nwabueta 2002, Okonokhua et al. 2007), with a large impact of the type of pollutant on the levels of these nutrients in the soil (Ogboghodo et al. 2004b). Contamination of the soil with engine oil may contribute to an increase of potassium in the soil (Obire and Nwabueta 2002, Wyszkowski and Ziółkowska 2008), which was reflected in our experiment. Petroleum products also caused a drop in pH, associated with an increased production of organic acids as a result of soil microbial metabolism, which confirmed the results of the study by Osuji and Nwoye (2007).‬

Despite the passage of 4 years from the soil contamination by diesel fuel and engine oil, the C_12_–C_36_ hydrocarbon levels were still several hundred times higher than those found in the uncontaminated soil, which proves their durability. Use of the ZB-01 biopreparation contributed to a 2- to 3.5-fold reduction in the levels of these substances. Our earlier monitoring of the decomposition of petroleum products in this experiment has shown that 2 years after contamination and biopreparation application, TPH levels in the soil contaminated with DF and EO were respectively 3 and 2 times lower than after 1 month from the soil contamination and in the objects subjected to bioremediation were about 2 times lower than in the soils without this treatment (Gospodarek et al.‬ 2016).‬ Comparison of the levels of petroleum products in a soil contaminated with petrol and a control soil, both with or without biopreparation, suggests that the contamination with petrol was neutralized by evaporation, and the obtained concentrations of organic compounds show naturally occurring organic substances in the soil (e.g. humic acids and others).‬

Unfavorable changes caused by petroleum substances in the soil cause changes in the growth and development of plants.‬ In our study, engine oil and diesel fuel had the most adverse effect on the morphological characteristics of winter wheat, although the plants proved to be relatively resistant to the soil contamination. Significant differences were recorded only in terms of plant height (a reduction of about 4–5%).‬ For comparison, broad bean (*Vicia faba* L.) grown in an analogous experiment on soil contaminated with engine oil or diesel fuel, had been characterized by a more than 30% lower number of leaves and mass of shoots compared to plants from uncontaminated soil, and the plants height on soil contaminated with engine oil was more than 40% lower than the control (Rusin et al. 2015).‬ As in the present experiment, there had been no significant negative effect of the petroleum products on the generative parts of the broad bean, i.e. the seeds.‬ Many authors emphasize the harmful effects of petroleum products on the growth and development of arable crops (Liste and Felgentreu 2006; Agbogidi et al.^2007^; Njoku et al.‬ 2012; Osuagwu et al.‬ 2013; Lopes and Piedade 2014). The unfavorable effects of engine oil and diesel fuel on arable crops can be explained by the negative impact of the hydrocarbons they contain on the transpiration and respiration of plants, as well as on the transport of nutrients through the cell membranes (Pezeshki et al.^2000^; Wake 2005). In addition, petroleum products cause a reduction in the levels of nutrients available for plants and disturbances in their uptake, which may cause problems with photosynthesis and a reduction in chlorophyll levels, and consequently lead to growth inhibition or death of the plants (Odjegba and Sadiq 2002).‬ Lack of a clear negative effect of petroleum-derived substances on winter wheat growth may indicate that this species can be used in areas exposed to this type of pollution.

Little is known about the impact on the growth of plants of the various techniques of natural bioremediation in soils contaminated with petroleum products.‬ For example, the addition of compost, bentonite and calcium oxide (Wyszkowski and Ziółkowska 2009a; Wyszkowski and Ziółkowska 2009b; Vouillamoz and Milke 2001) or poultry litter (Ogboghodo et al. 2004a) to soils contaminated with petroleum products may contribute to better growth and development of the plants. The microbial biopreparation used in our experiment caused only an increase in plant height in the soil contaminated with petrol.

The activity of antioxidant enzymes such as POD, SOD and CAT is often determined in studies on stress in plants caused by xenobiotics, mainly heavy metals.‬ These enzymes are involved in many processes, but above all are an important component of the antioxidant system in plants (Kafel et al. 2010; Boojar and Tavakkoli 2011).‬ An increase in POD activity in plants caused by the presence of heavy metals in the soil has been demonstrated by many authors (Dazy et al 2009, Kafel et al. 2010; Nadgórska-Socha et al. 2013a). Marti et al. (2009) also showed that soil contamination with sludge from a refinery containing hydrocarbons and heavy metals increased the activity of SOD and POD in alfalfa leaves. In the present experiment, diesel fuel and petrol caused an increase in POD activity, but at the same time resulted in a decrease in SOD activity. In turn, engine oil caused the reverse phenomenon, i.e. a decrease in the activity of POD and an increase in SOD activity. On the other hand, CAT activity was reduced by soil pollution with engine oil and diesel fuel.‬ The decrease of CAT activity was found by Tabassum et al. (2016) where the CAT assay showed a progressive decrease in values with increasing stress level due to the engine oil. Similar to our study, SOD activity increased with increasing concentrations of waste engine oil in *Mirabilis jalapa* (Tabassum et al. 2016).‬ It should be emphasized that the activity of the analyzed enzymes in plants depends on many factors, such as plant species, analyzed tissues and conditions of the experiments e.g. pollutant concentration (Marti et al. 2009; Tang et al. 2009, Gill and Tuteja 2010). The analysed petroleum products can have various effect on particular enzymes. SOD catalases the dismutation of superoxide to H_2_O_2_ and O_2_ and POD and CAT are involved in the decomposition of H_2_O_2_ to H_2_O. Moreover POD activity is suggesting as non-specific biomarker of environmental pollution. Engine oil had the most adverse effect on wheat plants because of the stress with slower protection against H_2_O_2_ decomposition. According to Dazy et al. (2009) and Papa et al. (2012) the plants possess antioxidant enzymes but these operate either unspecifically or depending on the nature and the level of contamination. It could be suggested that a synergy of abiotic and also biotic factors may have affected the plant responses. Based on the results further investigations should be performed to determine such phenomenon.

In a study by Dazy et al. (2008) plants grown on soil contaminated with heavy metals and polycyclic aromatic hydrocarbons exhibited changes in antioxidant enzyme activities (SOD and CAT).‬ *Oenothera biennis* exhibited the highest increase of SOD and CAT activity with a decrease in growth and biomass, which indicates oxidative stress. In *Matricaria recutita* also antioxidant enzymes activity was increased with increasing growth and biomass, suggesting that the cellular defense systems were able to cope with the pollutants and protected the cells. In our study we did not observe a significant decrease of plant mass, yet in one case (bioremediation of petrol-polluted soil) an increase in height of the plants was observed, which shows that the antioxidant system in the wheat protected the plants against the petroleum products.

The ZB-01 biopreparation resulted in the elimination of differences in POD activity from the soils contaminated with engine oil and diesel fuels, whereas SOD activity was reduced in plants exposed to engine oil pollution.‬ CAT activity under the influence of ZB-01 did not change in the case of pollution with diesel fuel and petrol, while it continued to decline in soil contaminated with engine oil.‬ Our findings are in agreement with the study of Balseiro-Romero et al. (2017). They also observed decrease of investigated antioxidant enzymes in plants inoculated with PGP (plant growth promoting bacteria) and growing in diesel contaminated soil, which means that microbial inoculation can provoke antioxidant enzymes activity decrease or decrease in plant oxidative stress, which could be translated into a better tolerance to soil contamination which was also reflected by better plant growth.

Low molecular weight antioxidants such as proline, ascorbic acid and glutathione neutralise free oxygen radicals.‬ Proline accumulation in plants is considered as an indicator of environmental stress caused by heavy metals, UV radiation, salinity or drought, etc.‬ Elevated levels of this amino acid increase tolerance to stress through osmoregulation, enzyme protection against denaturation, and stabilization of protein synthesis (Sharma and Dietz 2006, Szabados and Savouré 2009). An important role is also played by non-protein thiol groups (-SH), such as glutathione or cysteine, which are able to bind metal ions to form non-toxic complexes with them, thus becoming an important element in determining plant tolerance to xenobiotics (Sun et al.^2007^; Xu et al.‬ 2009).‬ Data regarding the impact of soil contaminants on the levels of -SH groups indicate a large variation in reactions, depending on the plant species and the type of pollutant (Kafel et al. 2010; Nadgórska-Socha et al. 2013a, 2013b). In the present experiment, only diesel fuel reduced the levels of non-protein thiol groups in winter wheat leaves, while engine oil and petrol led to their increase. These discrepancies can be explained by the fact that sulfur-containing molecules occur in many plant cells and fulfill a number of different functions, and can be independently regulated (Mishra et al. 2009).‬

Diesel fuel and petrol increased proline levels in winter wheat plants, which usually increase in response to stress caused by the presence of heavy metals or diesel, a regularity shown by many authors (Teklić et al. 2008; Tang et al. 2009; Kafel et al. 2010; Nadgórska-Socha et al. 2013a).‬ This may vary depending on the plant species, as heavy metals caused an increase in proline levels in *C. arenosa* leaves but a decrease in *P. lanceolata* leaves (Nadgórska-Socha et al. 2013b).‬ In our experiment, engine oil reduced proline levels in winter wheat leaves. John et al. (2009) also showed that with an increase in cadmium and lead levels in the soil, proline levels in *Brassica juncea* leaves dropped. Interestingly, large changes in the levels of both -SH and proline were observed when the ZB-01 biopreparation was used.‬ In each case (with the exception of petrol in relation to SH groups) the biopreparation caused a significant increase in the levels of both these antioxidants. ‬Free proline usually functions as a plant water stress indicator and petroleum products might restrain the root absorption of soil water and lead to higher free proline content what was observed in our experiment. Decrease in plant oxidative stress was contented rather with the increase demand for SH-rich compounds. It can be accounted for by activation of pathways involved in sulphur assimilation and cysteine biosynthesis (Marti et al. 2009; Tang et al. 2009).

The composition of plants is yet another factor that can be strongly modified by soil contamination with petroleum products.‬ Many authors emphasize that pollution with petroleum products may cause a reduction in nitrogen levels in plants (Wyszkowski et al.‬ 2004; Wyszkowski and Wyszkowska 2005), which coincides with the results of this experiment with respect to diesel fuel and petrol. In soil contaminated with petroleum substances, the nitrogen–carbon ratio is disturbed due to the presence of hydrocarbons. This contributes to the inhibition of many nitrogen reactions in the soil (regarding both mineral and organic forms of nitrogen), as well as to a reduction in the intensity of ammonification and nitrification (Adam and Duncan 2003; Kucharski and Jastrzębska 2005, which may explain the reduced levels of nitrogen in the presence of xenobiotics. Moubasher et al. (2015) showed that petroleum hydrocarbons usually do not cause a significant change in sulfur levels in the roots and shoots of *Bassia scoparia* (L.). However, in our experiment, contamination of the soil with either diesel fuel or petrol led to a decrease in sulfur levels in the plants.‬ These discrepancies result from the different plant species, as many authors emphasize that various species of crop plants are characterized by a different sensitivity to the presence of petroleum products in the soil (Wyszkowski and Ziółkowska 2009a). Petroleum products contribute to an increase in carbon levels in the soil, which results from their structure (Riffaldi et al.‬ 2006; Wyszkowski and Sivitskaya 2012).‬ This may contribute to elevated carbon levels in plants grown in soils contaminated with petroleum products, although it should be remembered that the main source of carbon for plants is atmospheric air, which is why no similar relationships were observed in the present experiment.‬

In the present experiment, diesel fuel caused a decrease in calcium levels in the plants. On the other hand engine oil and petrol increased the levels of Ca. Wyszkowski and Wyszkowska (2005) showed that engine oil caused a reduction in calcium levels in the aboveground parts of oats, with doses above 9 g kg^−1^ contributing to an increase in the levels of this nutrient in the aboveground parts of maize. Discrepancies regarding the influence of petroleum products on the calcium levels in plants have been demonstrated by many authors (Wyszkowski et al.‬ 2004, Wyszkowski and Ziółkowska 2009a, Wyszkowski and Ziółkowska 2009b). This indicates that effect of petroleum-derived substances on the content of this nutrient depends on the plant species and the type and dose of contamination. In a study by Wyszkowski and Wyszkowska (2005) and Wyszkowski and Ziółkowska (2009a), engine oil pollution caused an increase in potassium levels and a decrease in magnesium levels in oats and maize, which coincides with the results of our present experiment.‬ In literature there is no information on the influence of petroleum products on iron levels in plants.‬ Other soil contaminants such as heavy metals (zinc, nickel) may lead to an increase in iron levels (Gospodarek and Nadgórska-Socha 2010), which coincides with the results of this present experiment in the case of engine oil and diesel fuel.‬ Changes in the chemical composition of plants growing on soil contaminated with petroleum-derived substances may affect indirectly herbivores (Rusin et al. 2017) and consequently provide information about potential negative effects on further links of the food chain, i.e. for predators, parasitoids and also for consumers.

ANE may reveal abnormalities in the mutual relations between the components (Ostrowska and Porębska 2002). In the present study, the descending order of macroelements in total accumulated elements can be represented as N > S > K > Ca > Mg. The decrease especially in % of S and N was observed in ANE calculated for the plants treated by petroleum products, what is in line with the results for individual macroelements and other authors findings (Wyszkowski and Wyszkowska 2005; Wyszkowski and Ziółkowska 2009a). Ostrowska and Porębska (2002) found a higher value of ANE in bidens (*Bidens* sp.) grown in the field, in comparison with the same plant species from a landfill, which also showed a higher value of the sum of the trace elements. Increase of the value of ATE was calculated for plants treated by engine oil and diesel fuel. Higher values of ATE were also obtained for *Cardaminopsis arenosa* and *Plantago lanceolata* from metalliferous stands when compared to non-metalliferous sites (Nadgórska-Socha et al. 2015). Higher ATE values and decrease especially in % of S and N could cause lower growth [height of plants (Table 3)].

Available literature lacks data on the impact of bioremediation processes using microorganisms on nutrient levels in crop plants growing in soils contaminated with petroleum products.‬ The use of remediation techniques for contaminated soils, such as the addition of compost, bentonite and calcium oxide, shows that these activities can significantly modify the levels of macro- and microelements in plants (Wyszkowski and Ziółkowska 2009a). In the present experiment, the effect of the ZB-01 biopreparation varied and caused both an increase and a decrease in the levels of individual nutrients, depending on the type of xenobiotic.‬ Many authors emphasize that the effectiveness of bioremediation for soils contaminated with petroleum products depends on many factors, including the type of pollutant. Substances with a complex structure are usually less susceptible to this process (Van Hamme et al. 2003); the formation of toxic intermediates may also occur. The effectiveness of bioremediation also depends on soil moisture, temperature, pH and the bioavailability of substrates (Yerushalmi et al. 2003; Dindar et al.‬ 2013). All these factors may affect the composition of plants grown in soil subjected to bioremediation.‬

Available literature provides very little information on the impact of petroleum products on heavy metals in plants. Nwaichi et al. (2014) showed that petroleum products caused an increase in the levels of lead and zinc in the leaves of *Vernonia amygdalin, Talinum triangulare, Manihot esculenta* and *Xanthotosoma sagittifolium*. Similar dependencies in the present experiment were found in the case of engine oil, which additionally led to an increase in the levels of manganese and cadmium in the aboveground parts of winter wheat.‬ Rusin et al. (2015) found that oil products caused an increase in the levels of lead and manganese in broad bean leaves, which is also partly consistent with the results of the present experiment. The influence of petroleum products on the levels of heavy metals, however, is variable and depends on the type of component being analyzed, the dosage and type of compound used, and the part of the plant being analyzed, as demonstrated by Rusin et al. (2017).‬ Petroleum products modify the levels of heavy metals in the soil, which can also indirectly affect their levels in plants (Santos-Echeandia et al. 2008). The absorption of heavy metals by plants is usually positively correlated with the amount in the soil in which they grow (LeCoultre 2001). Many authors have shown that petroleum products cause an increase in the levels of cadmium, lead or manganese in the soil (Ujowundu et al. 2011; Wyszkowski and Sivitskaya 2012).‬ An increase in the content of heavy metals in plants may cause them to lose their value for consumption and fodder purposes, which indicates the need to monitor their content when we introduce plants (e.g. winter what) for this purpose on areas exposed to pollution with petroleum products.

Nanekar et al. (2015) showed that the introduction of additional microorganisms into soils contaminated by petroleum products (bioaugmentation) reduced the hydrocarbon level of the oils in the soil, and also reduced the levels of some heavy metals, e.g. lead.‬ This is due to the ability of microorganisms to adapt to adverse conditions and use harmful compounds for their growth and development, to increase the rate of compound degradation.‬ Similar patterns were also demonstrated by other authors (Mukherjee and Bordoloi 2011). Biopreparation ZB-01 used in this experiment caused a decrease in lead levels in the plants from soil contaminated with engine oil and a decrease in manganese levels in the plants from soil contaminated with diesel fuel. However, it also contributed to an increase in the levels of some heavy metals such as: zinc in the plants from soil contaminated with petrol.‬

## Conclusions‬

1. Petroleum products contributed to changes in the physicochemical properties of the soil, causing an increase in acidification, a decrease in the levels of available phosphorus and an increase in the available levels of potassium and total carbon.‬

2. The studied winter wheat was relatively resistant to soil contamination with petroleum products, and did not show a significant impact on the morphological characteristics of the plants when growing in the polluted soil.‬

3. The petroleum products significantly affected the activity of antioxidant enzymes and the levels of antioxidants in the plants.‬ The general markers of soil contaminated with diesel fuel and petrol were POD activity and proline levels in the leaves. These parameters seem to be universal antioxidant defense factors in exposure to petroleum products.‬

4. Petroleum products significantly modified the levels of nutrients and heavy metals in the plants, depending on the type of substance and the analyzed component.‬

5. Use of the ZB-01 biopreparation in soils contaminated with petroleum products showed a variable effect depending on the type of pollutant and the analyzed feature.‬ In general, it caused an increase in the levels of proline and -SH groups and an increase in the levels of carbon and calcium in the plants. However, it had no effect on the morphological characteristics of plants.‬
